# Validating measures of stigma against those with mental illness among a community sample in Kilifi Kenya

**DOI:** 10.1017/gmh.2022.26

**Published:** 2022-06-03

**Authors:** Mary A. Bitta, Judy Baariu, Elias Fondo, Symon M. Kariuki, Belinda Lennox, Charles R. J. C. Newton

**Affiliations:** 1 Clinical Research-Neurosciences, KEMRI/Wellcome Trust Research Programme, Centre for Geographic Medicine Research (Coast), Kilifi, Kenya; 2 Department of Psychiatry, University of Oxford, Oxford, UK; 3 Department of Psychiatry, Kilifi County Hospital, Kilifi, Kenya

**Keywords:** CAMI III, Kenya, Kilifi, Kiswahili, MAKS, mental disorders, psychometrics, RIBS, stigma

## Abstract

**Background:**

Stigma against persons with mental illness is a universal phenomenon, but culture influences the understanding of etiology of mental illness and utilization of health services.

**Methods:**

We validated Kiswahili versions of three measures of stigma which were originally developed in the United Kingdom: Community Attitudes Toward the Mentally Ill Scale (CAMI), Reported and Intended Behaviors Scale (RIBS) and Mental Health Awareness Knowledge Schedule (MAKS) and evaluated their psychometric properties using a community sample (*N* = 616) in Kilifi, Kenya.

**Results:**

Confirmatory factor analysis confirmed the one-factor solution for RIBS [root mean-squared error of approximation (RMSEA) < 0.01, comparative fit index (CFI) = 1.00, Tucker–Lewis index (TLI) = 1.01] and two-factor solution for MAKS (RMSEA = 0.04, CFI = 0.96, TLI = 0.95). A 23-item, three-factor model provided the best indices of goodness of fit for CAMI (RMSEA = 0.04, CFI = 0.90, TLI = 0.89). MAKS converged with both CAMI and RIBS. Internal consistency was good for the RIBS and acceptable for CAMI and MAKS. Test–retest reliabilities were excellent for RIBS and poor for CAMI and MAKS, but kappa scores for inter-rater agreement were relatively low for these scales. Results support validity of the original MAKS and RIBS scale and a modified CAMI scale and suggest that stigma is not an enduring trait in this population. The low kappa scores are consistent with first kappa paradox which is due to adjustment for agreements by chance in case of marginal prevalence values.

**Conclusions:**

Kiswahili versions of the MAKS, RIBS and a modified version of the CAMI are valid for use in the study population. Stigma against people with mental illness may not be an enduring trait in this population.

## Background

Stigma in mental illness is a complex construct that reflects problems in knowledge (ignorance), attitude (prejudice) and behavior (discrimination) toward people with mental disorders, their families and caregivers (Goffman, 1963; Link and Phelan, 2001; Thornicroft *et al*., 2007; Fox *et al*., 2018). Stigma against people with mental illnesses is well established as a major contributing factor for poor treatment and disease outcomes. Poor outcomes associated with stigma include low job prospects (Luciano and Meara, 2014), poorer prospects of social relationships such as marriage (Breslau *et al*., 2011), increased risk for comorbidities (Nock *et al*., 2010), lower quality of life and premature mortality compared to the general population (Evans *et al*., 2007). Globally, less than half of the people with mental disorders receives minimally adequate and evidence-based care (Patel *et al*., 2010) in part because of stigma. These poor outcomes are partly attributed to ignorance about the etiology of mental disorders as well as prejudicial attitudes and discriminatory behavior by the general public and care providers (Henderson *et al*., 2014). Conceptual models of stigma posit that poor health outcomes for people with mental illness occur through negative emotional responses and behaviors such as fear of seeking help (Clement *et al*., 2015; Luitel *et al*., 2017; Zewdu *et al*., 2019).

A recent review found over 400 measures of stigma in mental illness, majority of which were unvalidated in the contexts in which they were used (Fox *et al*., 2018). In the last two decades, new measures were developed at an approximate rate of 36 measures annually perhaps due to the nuances in the conceptualization of the construct of stigma (Fox *et al*., 2018). The rapid increase in the development and use of unvalidated measures of stigma may have saturated the need for new scales, and future studies should focus on validating and improving available measures. In this study, we validated the Mental Awareness Knowledge Schedule (MAKS) (Evans-Lacko *et al*., 2010), Reported and Intended Behaviours Scale (RIBS) (Evans-Lacko *et al*., 2011) and Community Attitudes Toward the Mentally Ill scale (CAMI) (Taylor and Dear, 1981) in a community sample in Kilifi Kenya. The MAKS, RIBS and CAMI assess knowledge, behavior and attitude, respectively. These tools have been validated in community samples from high-income settings and results suggest that the original one- and two-factor structures of the RIBS and MAKS, respectively, are valid in the assessment of stigma (Garcia *et al*., 2017) but the original four-factor structure of the CAMI does not hold in community samples (Brockington *et al*., 1993; Wolff *et al*., 1996; Garcia *et al*., 2017). However, there are no data on the validity of these tools in the assessment of mental health stigma in Kenya.

The MAKS (Evans-Lacko *et al*., 2010) is a 12-item questionnaire that measures a heterogenous group of items relating to mental health knowledge. It is divided into two parts that measure stigma-related mental health knowledge and knowledge about specific mental illness conditions. Previous validation studies reported poor internal consistency of the MAKS scale (Evans-Lacko *et al*., 2010; Pingani *et al*., 2019). However, because people's knowledge may be domain specific, internal consistency is not a relevant measure of this tool's utility. The RIBS scale (Evans-Lacko *et al*., 2011) is an 8-item scale that measures the prevalence of observed behavior (items 1–4) and intended behavior (items 5–8). Previous studies have found good psychometric properties of the RIBS scale (Pingani *et al*., 2016). CAMI (Taylor and Dear, 1981) is a 40-item scale designed to measure attitudes of the general population toward people with mental illness. It comprises four domains (each with 10 questions): authoritarianism, benevolence, social restrictiveness and community mental health ideology. Each domain has 10 questions each. Authoritarianism reflects the view that people with mental illness are inferior and that they should be handled using force or threats. Benevolence involves a sympathetic view of patients based on religious and humanistic principles. Social restrictiveness is a view that the mentally ill are a threat to society. Community mental health ideology is the idea that the whole community should work together through a variety of community resources to assist patients.

In low- and middle-income countries, these tools have been used for general populations (Abi Doumit *et al*., 2019), community samples (Girma *et al*., 2013; Reta *et al*., 2016; Basu *et al*., 2017; Hartini *et al*., 2018; Abi Doumit *et al*., 2019; Niedzwiedz, 2019; Tesfaye *et al*., 2020; Potts and Henderson, 2021) and special populations such as health professionals and medical students (Mutiso *et al*., 2017; Siqueira *et al*., 2017; Fekih-Romdhane *et al*., 2021). However, they have also not been validated in most of these settings yet studies that have validated the tools in community samples show that while their original factor structures are retained in some samples (Abelha *et al*., 2015), they do not hold in others (Brockington *et al*., 1993; Wolff *et al*., 1996; Abelha *et al*., 2015). In Kenya, only one study has used all three tools to evaluate effectiveness of an anti-stigma social marketing campaign, but this study did not translate the tools to Kiswahili, which is Kenya's lingua franca, and it did not conduct any psychometric analysis to validate the tools for the population on which they were used (Potts and Henderson, 2021).

This study validated and evaluated the psychometric properties of the Kiswahili versions of the CAMI, MAKS and RIBS scales, in a community sample in Kilifi Kenya. This analysis was part of a preliminary phase of a study that will provide contextually valid stigma tools for the measurement of the effectiveness of the *Difu Simo* Mental Health Awareness Campaign (Collaborators, 2019; https://difusimo.org), which will address mental health stigma in Kilifi county.

## Methods

### Study setting

This study was conducted at the Kilifi County Hospital (KCH) which is the largest teaching and referral hospital in Kilifi. KCH is in Kilifi township, the administrative and commercial headquarters of Kilifi. Kilifi county is predominantly rural and is located along the coast of the Indian Ocean with a population of ~1.5 million residents (KNBS, 2019). The main economic activities are agriculture, fishing, tourism and small-scale trade. Kiswahili language is Kenya's lingua franca. The burden of common mental and neurological disorders in this population is high (Ngugi *et al*., 2013; Kariuki *et al*., 2017; Kind *et al*., 2017) and there is evidence of stigma toward people with these disorders in Kilifi (Mbuba *et al*., 2012).

### Participants

Between August 2020 and June 2021, a community sample of people ⩾18 years, living within a 25-km radius of KCH was recruited. Distance restriction was applied to minimize participant movement in adherence to the government's COVID-19 guidelines (MOH, 2020). The study was advertised through local government administrators and participant recruitment was sequential on a first-come first served basis until the desired sample size was achieved. Lack of fluency in Kiswahili language and severe mental or neurological disability and incapacity to consent or participate as verified by a clinician and patient's ability to provide informed consent were the exclusion criteria. Based on expected values of the confirmatory factor analysis (CFA) from existing literature (Brockington *et al*., 1993; Wolff *et al*., 1996; Pingani *et al*., 2019; Tong *et al*., 2020) we aimed for a sample size >600. This sample size would be well powered for other analyses such as reliability testing which requires fewer samples (*N* = 100) to detect acceptable correlations (>0.3) with >0.80 accuracy.

In addition, we collected self-reported sociodemographic data on participants' experience with mental illness or epilepsy either as caregivers or patients. Qualitative research from the study setting indicates that epilepsy is viewed as a mental rather than neurological illness and patients are likely to face the same stigma as those with mental illnesses (Bitta *et al*., 2020). Caregivers were defined as primary care providers for persons with either mental illness or epilepsy. Medical records were used to verify those who identified as patients. Face to face interviews were conducted by trained raters. After the first interview, participants were provided with additional information about the *Difu Simo* awareness campaign. They were given vignettes about common mental disorders, fliers and links to the project's website and social media pages.

### Measures

English versions of the three scales were translated to the Kiswahili language and validated. Translation to Kiswahili followed the World Health Organization's guidelines of forward translation, expert panel back translation, pretesting, cognitive interviewing and testing of the final version (WHO, 2009). Forward translation from English to Kiswahili was done by two independent translators fluent in the Kiswahili language. Back translation of the Kiswahili tools was done by three clinicians. We then invited the first 20 participants who enrolled for the study to pretest the tools and provide feedback on tool wording. This feedback was then used to create the final version of the tools (online Supplementary files 1–3). Changes included revision of phrases to make contextual sense for instance question *n* of the CAMI scale ‘Increased spending on mental health services is a waste of tax dollars’ was revised to read ‘Increased spending on mental health services is a waste of tax money’ since Kenya's currency is shillings.

For all the three scales, items were originally coded on an ordinal scale of 1 to 5 where 1 represented strongly disagree and 5 represented strongly agree. Neutral responses were scored as 3. Summated scores were calculated by adding the points obtained for each question. Higher scores indicated higher levels of knowledge for the MAKS, favorable attitudes toward people with mental illness for the CAMI and favorable intended behaviors for the RIBS. For the MAKS, items 6, 8 and 12 were reverse coded to ensure consistency with direction of the right responses for other questions.

### Statistical analysis

All data were analyzed using STATA (Version 15). Two-way analysis of variance or Mann–Whitney *U* test were used where appropriate to compare: (i) the distribution of sociodemographic characteristics between males and females and across the different groupings of experience with mental illness or epilepsy and (ii) to compare the composite scores between groups. All items were treated as continuous variables.

### Validity

To evaluate the internal validity of all the scales we first conducted CFA to validate (i) original structure as proposed by the tool developers and (ii) alternative structures available in literature from community samples (Brockington *et al*., 1993; Wolff *et al*., 1996). Where the original structure and structures suggested in literature could not be established, we investigated alternative structures by conducting exploratory factor analysis (EFA) where the sample was randomly split into two equal sizes and then EFA was conducted using principal factor analysis with Promax rotation. The Kaiser–Meyer–Olkin (KMO) measure of sampling adequacy and Bartlett's test of sphericity were used to determine the factorability of the scales. A KMO value of <0.5 value was acceptable. To determine the number of factors to extract, we first conducted principal factor analysis and then used the STATA command ‘fapara’ to conduct parallel analysis. Parallel analysis was conducted to determine the number of factors to extract. Additionally, the following criteria were applied to extract the factors (Norris and Lecavalier, 2010): (i) each factor contained only items that explained ⩾10% of the factor's variance, (ii) factors had at least three items loading with a factor loading ⩾0.32, (iii) only items that did not cross load on multiple factors with similar magnitudes were extracted and (iv) factors were interpretable in a contextually sensible way.

We then used CFA to validate the alternative structure using structural equation modeling to produce standardized factor loadings and goodness of fit measures, specifically the Tucker–Lewis index (TLI), the comparative fit index (CFI) and the root mean-squared error of approximation (RMSEA). An RMSEA ⩽ 0.06 and CFI and TLI ⩾ 0.95 were interpreted as good fits with RMSEA ⩽ 0.08 and CFI/TLI ⩾ 0.90 considered acceptable (Hu and Bentler, 1999). We analyzed a polychoric correlation matrix and used diagonally weighted least squares to estimate model parameters.

To measure convergent validity, we used Pearson's correlation coefficient to test the hypothesis, from previous studies that the summated MAKS scale scores were positively related to both the summated RIBS and CAMI scores (Garcia *et al*., 2017).

### Reliability

We used the Stata command ‘kappaetc’ to calculate inter-rater reliability. This command produces the following coefficients: percent agreement, Brennan and Prediger's coefficient, Cohen's kappa (*κ*), Scott/Fleiss' kappa, Gwet's AC and Krippendorff's alpha. For all the coefficients, a score of 1 suggested perfect agreement while a score closer to 0 suggested poor agreement between two independent interviewers. Inter-rater interviews were conducted approximately 1 h apart. Using the intra-class correlation coefficients, we measured test–retest reliability by comparing the reliability of means approximately 2 weeks apart. We used the McDonald's omega (*ωt*) to measure the internal consistency of all the scales because it performed better in a Monte Carlo simulation even when the item distributions were skewed (Trizano-Hermosilla and Alvarado, 2016).

## Results

### Sociodemographic characteristics of participants

Table 1 summarizes the sociodemographic characteristics of the participants. In total, we recruited 624 participants, but eight participants had incomplete data in at least one of the tools and were excluded from analysis. Therefore, our final sample size was 616 participants, of whom 313 (50.8%) were female. Participants' mean age was 37.4 (s.d. = 14) with no significant differences between men and women (*p* = 0.85) and the age range was 18–92 years. In total, 196 (31.8%) participants had lived experience with mental illness or epilepsy, either as patients (*n* = 72, 11.7%) or caregivers (*n* = 124, 20.1%). There were significantly more male patients with epilepsy (*p* = 0.03), while caregivers of people with epilepsy or mental illness were predominantly female (*p* < 0.01). Females had higher proportions of participants with no formal education (*p* < 0.01), while there were more male participants with secondary (*p* < 0.01) and tertiary education (*p* < 0.01).

**Table 1.** Sociodemographic characteristics of study participants

Participant characteristic	Overall, *n* = 616	Male, *n* = 303	Female, *n* = 313	*p* value for significance in proportion differences
Mean age (s.d.)	37.4 (14.0)	37.5 (15.2)	37.3 (12.7)	0.85
Level of education, *n* (%)				
None	56 (9.1)	13 (4.3)	43 (13.7)	<0.01
Primary	243 (39.5)	103 (34.0)	140 (44.7)	0.01
Secondary	221 (35.9)	127 (42.0)	94 (30.0)	<0.01
Tertiary	96 (15.6)	60 (19.8)	36 (11.5)	<0.01
Experience with mental illness, *n* (%)				
Has mental illness	30 (4.9)	10 (3.3)	20 (6.4)	0.07
Has epilepsy	42 (6.8)	27 (8.9)	15 (4.8)	0.04
Caregivers of person with mental illness or epilepsy	124 (20.1)	45 (14.9)	79 (25.2)	<0.01

### Distribution of item responses

Results for all summated scores are provided in Table 2. The summated mean score of the RIBS scale was 15.6 (s.d. = 4.8) out of a possible 20 and there was no difference (*p* = 0.76) in the summated mean scores between those with mental illness (15.6, s.d. = 4.7) experiences and those without (15.7, s.d. = 5.0). There were no significant differences in the distribution of scores by sex or experience with mental illness but there was a significant difference by levels of education (*p* = 0.00) as shown in online Supplementary Table S1. Over 50% of the respondents selected the response ‘strongly agree’ in questions 5, 7 and 8 of the RIBS scales. For question 6 ‘In future, I would be willing to work with someone with a mental health problem’, 45.1% of the respondents selected the ‘strongly agree’ response as shown in online Supplementary Table S2.

**Table 2.** Summated and subscale mean scores and standard deviations of the original CAMI, MAKS and RIBS scales by sex and experience with mental illness

	Overall	Male, *n* = 303	Female, *n* = 313	Has mental illness, *n* = 30	Has epilepsy, *n* = 42	Caregivers, *n* = 124	No experience with mental illness, *n* = 420
CAMI							
Summated mean score, mean (s.d.)	71.0 (7.3)	69.9 (7.3)	72.2 (7.2)	73.3 (7.2)	73.2 (8.7)	72.1 (7.0)	70.3 (7.2)
CAMI factor 1 (maximum possible score of 65)	28.6 (6.8)	27.8 (6.9)	29.4 (6.6)	29.6 (6.9)	30.9 (7.8)	29.0 (6.1)	28.2 (6.8)
CAMI factor 2 (maximum possible score of 35)	31.2 (3.1)	31.0 (3.0)	31.4 (3.3)	31.9 (3.4)	31.1 (3.5)	31.6 (3.2)	31.0 (3.0)
CAMI factor 3 (maximum possible score of 15)	11.3 (2.6)	11.1 (2.5)	11.4 (2.6)	11.8 (2.2)	11.2 (2.4)	11.6 (2.6)	11.1 (2.6)
MAKS							
Summated score	43.1 (4.4)	43.1 (4.5)	43.1 (4.3)	44.7 (5.1)	43.1 (4.0)	43.8 (3.9)	42.8 (4.5)
Mental health knowledge items	22.6 (3.1)	22.5 (3.2)	22.7 (3.0)	24.2 (2.3)	23.1 (3.0)	23.0 (3.0)	22.3 (3.2)
Mental illness condition knowledge	20.5 (2.8)	20.6 (2.9)	20.4 (2.8)	20.5 (4.0)	20.0 (2.9)	20.7 (2.7)	20.5 (2.8)
RIBS							
Summated score, mean (s.d.)	15.6 (4.8)	15.8 (4.6)	15.4 (5.1)	14.7 (4.9)	16.0 (5.1)	15.9 (5.1)	15.6 (4.8)

The mean MAKS score was 43.1 (s.d. = 4.4) out of a possible 60 and those with experience in mental illness had significantly higher levels of knowledge than those without (42.8, s.d. = 4.5 *v*. 43.8, s.d. = 4.1, *p* = 0.01). Sixty five percent of respondents strongly disagreed with item 6 ‘Most people with mental health problems go to a healthcare professional to get help’. Schizophrenia was recognized as a mental disorder by 83.4% of the participants, followed by drug addiction (57.5%), then depression and bipolar disorder (49.2% each) as summarized in online Supplementary Table S3. There was no significant between group difference by sex (*p* = 0.99) but there were significant differences by level of education (*p* = 0.02) and experience with mental illness (*p* = 0.01) (online Supplementary Table S1).

The mean CAMI score was 71 (s.d. = 7.3). People with mental illness experiences had significantly better attitudes than those without (196: 72.6, s.d. = 7.4, *v*. 70.3, s.d. = 7.2, *p* < 0.01). Results of the frequency distributions for each of the 40 CAMI items are shown in online Supplementary Table S4. Women had significantly higher scores in factor one scores (*p* = 0.00) compared to men. Additionally, level of education had a significant association with overall scores for factor one (*p* = 0.00) but not factor two (0.65) or three (0.60) as indicated in online Supplementary Table S1.

### Reliability

As summarized in Table 3 RIBS [*ωt* = 0.87, 95% confidence interval (CI) 0.84–0.89] had a good internal consistency while CAMI (*ωt* = 0.78, 95% CI 0.76–0.81) and MAKS (*ωt* = 0.70, 95% CI 0.67–0.74) had acceptable internal consistencies.

**Table 3.** Reliability of the stigma scales with 95% CIs

Coefficient	RIBS	MAKS	CAMI
Percent agreement	0.96 (0.94–0.97)	0.96 (0.95–0.97)	0.96 (0.95–0.097)
Brennan and Prediger	0.79 (0.72–0.86)	0.80 (0.76–0.84)	0.79 (0.75–0.83)
Cohen/Conger's kappa	0.77 (0.68–0.85)	0.35 (0.20–0.50)	0.51 (0.38–0.63)
Scott/Fleiss' kappa	0.76 (0.69–0.84)	0.36 (0.21–0.51)	0.48 (0.36–0.60)
Gwet's AC	0.88 (0.83–0.93)	0.83 (0.79–0.87)	0.81 (0.77–0.85)
Krippendorff's alpha	0.77 (0.69–0.85)	0.33 (0.18–0.48)	0.46 (0.34–0.59)
McDonald's omega (*ωt*)	0.87 (0.84–0.89)	0.70 (0.67–0.74)	0.78 (0.76–0.81)

Inter-rater reliability testing was conducted on 161 (26.1%) participants. Data on percentage agreement and coefficient scores are presented in Table 3. Percentage agreement was high (>96%) for all the scales while kappa coefficients ranged from 0.4 (95% CI 0.2–0.5) using Cohen's kappa to 0.9 (95% CI 0.8–0.9) using Gwet's coefficient. Test–retest reliability was assessed for 59 participants (9.6%). It was excellent for RIBS (Intracluster correlation coefficient (ICC) = 0.81, 95% CI 0.71–0.88) and poor for CAMI (ICC = 0.39, 95% CI 0.05–0.60) and MAKS (ICC = 0.19, 95% CI −0.26 to 0.47).

### Validity of the stigma scales

The original one- and two-factor structure for the RIBS (RMSEA < 0.01, CFI = 1.00, TLI = 1.01) and MAKS (RMSEA = 0.04, CFI = 0.96, TLI = 0.95), respectively, were established for this sample. The original four-factor structure for the CAMI could not be established (Table 4). We found two studies that validated the CAMI using community samples (Brockington *et al*., 1993; Wolff *et al*., 1996). Both studies proposed a three-factor structure, but these structures could not be established in our sample. We therefore conducted EFA to establish an alternative factor structure (online Supplementary Table S5). The KMO was acceptable (0.83) and the determinant of correlation matrix was significant (*p* < 0.01) indicating acceptable levels of correlation between items. Results of the parallel analysis proposed a six-factor structure, however three of the factors did not meet all our criteria, and we therefore retained three factors and a total of 23 questions. These factors cumulatively explained 86.1% of the variance. Online Supplementary Table S1 shows the factor loadings for each of the original 40 items. Factor one's items corresponded with the pro-authoritarianism, pro-social restrictiveness and anti-community mental health initiative domains of the original four-factor structure. Factor two corresponded with pro-benevolence, anti-authoritarianism and pro-community mental health initiative domains. Factor three corresponded with anti-social restrictiveness, anti-authoritarianism and pro-community mental health initiative domains.

**Table 4.** Comparisons of model fits for the three stigma scales, *n* = 616

Model	χ^2^	df	*p* value	RMSEA	CFI	TLI
CAMI original four-factor model	3025.71	5071.52	<0.01	0.07	0.47	0.43
Wollf's model for CAMI	863.02	1822.68	<0.01	0.08	0.57	0.55
Brockington's model for CAMI	269.48	1013.31	<0.01	0.07	0.79	0.73
CAMI modified three-factor model	492.31	3024.85	<0.01	0.04	0.90	0.89
RIBS one-factor model	0.218	1190	<0.01	0.00	1.00	1.01
MAKS two-factor model	100.23	1341.71	<0.01	0.04	0.96	0.95

Acceptable indices of goodness of fit were <0.06 for RMSEA and >0.90 for CFI and TLI.

χ^2^, chi-squared; RMSEA, root mean square error of approximation; df, degrees of freedom; CFI, comparative fit index; TLI, Tucker–Lewis index.

Summated MAKS score was positively related to both the summated RIBS (coefficient = 0.2, *p* < 0.05) and CAMI scores (coefficient = 0.09, *p* < 0.05). Each of the CAMI factors were also correlated with the MAKS summated scores and RIBS summated scores as summarized in Table 5.

**Table 5.** Convergent validity of the three stigma scales

	CAMI factor 1	CAMI factor 2	CAMI factor 3	CAMI summated scores	RIBS summated scores	MAKS general knowledge	MAKS mental illness knowledge	MAKS summated scores
CAMI factor 1	1							
CAMI factor 2	−0.22*	1						
CAMI factor 3	−0.09*	0.23*	1					
CAMI total	0.80*	0.30*	0.37*	1				
RIBS total	−0.26*	0.13*	0.31*	−0.08*	1			
MAKS general	−0.11*	0.18*	0.34*	−0.10*	0.20*	1		
MAKS illness	−0.04	0.05*	0.15*	0.03	0.09*	0.08	1	
MAKS total	−0.10*	0.16*	0.34*	0.09*	0.20*	0.76*	0.70*	1

*p < 0.05.

## Discussion

This study validated and evaluated the psychometric properties of the CAMI, RIBS and MAKS scales in a community sample in Kilifi Kenya. The indices for goodness of fit for the original RIBS and MAKS scales were excellent, so are applicable in our settings. Internal consistency was good for RIBS, acceptable for CAMI and low for MAKS, indicating that items are less related in the former scale and should be evaluated in future studies. The original one- and two-factor structures for the RIBS and MAKS, respectively, were retained in this sample, underscoring cross-cultural invariance of the scale. CAMI fitted into a three-factor structure that comprised of 23 out of the original 40 questions, suggesting that a shorter version may better fit this population, where literacy levels are low. The three scales likely measure a common construct of stigma since MAKS showed good convergent validity with both the RIBS and CAMI scales. These findings together suggest that the Kiswahili versions of the original RIBS and MAKS scales can be used to measure stigma in Kilifi.

Our sampling technique was not entirely random since we used a community sample that lived around KCH, and the population around this area is relatively urban compared to the rest of the county. The study population is part of a health and demographic surveillance system (Scott *et al*., 2012) where anti-stigma interventions have been conducted over the years (Ibinda *et al*., 2014; Collaborators, 2019) and this may have contributed to the higher levels of knowledge. Indeed, compared to other studies (Pingani *et al*., 2016; Pingani *et al*., 2019) item responses were skewed toward higher scores meaning that participants reported higher levels of knowledge, better attitudes and better intended behaviors. Additionally, participant recruitment, like in any survey, relied on cooperative participants who were likely to have more tolerant attitudes. Acquaintance to mental illness by patients or caregivers contributes to willingness to participate in mental health research (Crisp and Griffiths, 2014) and higher levels of tolerance toward the mentally ill (Brockington *et al*., 1993). This may explain the overall high scores in our study in which 31% of the participants had experiences with mental illness.

Convergent validity measures showed that there was significant positive correlation between the summated MAKS and both the summated CAMI III and RIBS scores which is similar to a French study that examined the correlation between the RIBS and MAKS instruments (Garcia *et al*., 2017). This indicates that the measures are theoretically related, which provides a rationale for using them together to measure the construct of stigma. However, we did not examine overlap between components of the different sub-scales and well-powered future studies should examine this to create a concise measure of stigma that taps on unique properties from each of the sub-scales. Internal consistencies of the CAMI and RIBS scales were acceptable, suggesting that their items were related to each other and were indeed measuring the stigma construct they intended to measure. The MAKS had a low internal consistency, which was comparable to that of the original sample on which the tool was developed. However, as explained by the tool developers, MAKS was not intended to function as a scale and the reliability values should only be used to explain trends in responses (Evans-Lacko *et al*., 2010).

Although percent agreement between raters was high for all the scales, the kappa coefficients were acceptable for the RIBS, but low for the MAKS and CAMI scales. This phenomenon, referred to as the first kappa paradox (Feinstein and Cicchetti, 1990) was not surprising, since we expected that there would be a skewed distribution of the frequencies of responses because this population has been exposed to anti-stigma interventions (Collaborators, 2019). This finding should not be interpreted as a limitation but rather as a logical consequence of the model's attempt to correctly interpret agreement, adjusted for chance. The test–retest reliabilities for the CAMI and MAKS scores were poor, similar to findings of the original scales (Taylor and Dear, 1981; Evans-Lacko *et al*., 2010) and of other validation studies (Garcia *et al*., 2017). After the original data collection, participants were exposed to anti-stigma activities (Collaborators, 2019) which may have affected the retest responses. Given the acceptable internal consistencies of all the scales, the test–retest finding suggests that attitudes and knowledge are not enduring traits in this population. Similar findings have been observed in other parts of Kenya where there were no ongoing anti-stigma interventions (Potts and Henderson, 2021); taken together these findings suggest that sustained awareness campaigns may be useful in this setting.

In the CFA, we found that a modified three-factor model, two-factor model and one-factor model for the CAMI, MAKS and RIBS scales, respectively, should be favored for the Kiswahili versions. The two- and one-factor model solutions for the MAKS and RIBS scales are similar to those of French and Portuguese validation studies (Garcia *et al*., 2017; Silva Ribeiro *et al*., 2021), underscoring configural invariance of these scales. Compared to our study, the French study had better indices of goodness of fit for the MAKS scale probably because it was conducted among nursing students, whose understanding of mental health is much better compared to our community sample. RIBS- and MAKS-factor structures corresponded perfectly with the original scales of a one- and two-factor structure, respectively, suggesting that the translated Kiswahili versions of the original scales can be used for this population.

CAMI's modified three-factor model with 23 items performed best, and it is possible this shorter version works better in this population because of low literacy levels. Although our items did not perfectly correspond with those of Brockington (Brockington *et al*., 1993) and Wolff (Wolff *et al*., 1996) who validated this tool in community samples, our three-factor structure suggested three levels of tolerance similar to their studies. First, an authoritarian attitude that reinforces isolation of people with mental illness, second a tolerant attitude and lastly a sympathetic attitude toward those with mental illness. This finding is perhaps timely as it may inform implementation of Kenya's 2021–2025 Mental Health Action plan, that includes among other things, plans to increase mental health literacy and reduce stigma (HealthTaskforce, 2021).

We found significant associations between some sociodemographic variables and stigma scores, using simple tests of comparison which suggested that further multivariable analysis is required to determine the sociodemographic correlates of stigma scores. These analyses will be conducted and presented as a separated manuscript, as part of a quantitative evaluation of the *Difu Simo* campaign.

### Strengths and limitations

The study had some strengths. First, persons with experience in mental illness or epilepsy were included, which allowed for comparison of stigma measures among those with and without mental health experiences. Second, the relatively large sample size allowed for robust validation models. Lastly, inclusion of all three tools allowed for measures of convergence among the three constructs of stigma, providing insights into their relationships in this setting. Our study combined EFA and CFA, a practise that is encouraged when the aim of a study is to identify latent structures that can be generalized and are clinically useful (Schmitt *et al*., 2018). Simulation studies have found that use of single-factor analytic approaches create challenges such as models overfitting data and producing errors and noise resulting in factors that are not clearly interpretable and hence cannot inform theory development (Montoya and Edwards, 2021; Greene *et al*., 2022).

This study also has limitations. We did not validate the short version of the CAMI scale independently. We only administered the questionnaire in its long form and hence we could not calculate the correlation between the long form and the short form because as described by Smith *et al*. (2000) this method would lead to an overestimation of the correlation between the two forms. Additionally, factors such as sample characteristics, linguistic adaptation and data-driven decisions in the analysis and interpretation may have contributed to the poor fit of the full version of the CAMI scale. Therefore, we recommend that authors should use the full 40-item versions as a start and conduct validation studies in their study populations. For Kilifi, where this study was conducted, future validation steps will include subjecting the excluded 17 questions to cognitive debriefing and cultural equivalence to assess the clarity of instructions and the comprehensibility of the content. Additional steps will include content equivalence exercises which will involve expert evaluation of relevance of contents (Sousa and Rojjanasrirat, 2011 #3).

Our study did not systematically examine essential unidimensionality of each scale, that is whether the set of items measured only one attribute or dimension. We recommend that future studies examine this assumption to improve interpretability overall scores, such as those used in our construct validity models. Lastly, we cannot quantify the extent to which social desirability influenced the results.

## Conclusions

The Kiswahili version of the original MAKS and RIBS scales can be used to assess knowledge and behavior in Kilifi. CAMI-23 scale may be best suited for this population, but further studies are required to validate it against other scales measuring similar construct of knowledge. Despite earlier anti-stigma interventions of epilepsy in the area, problem of stigma is still substantial in this area. The constructs of stigma are not enduring traits in this population suggesting that anti-stigma interventions have a place in this setting.

## Supplementary material

For supplementary material accompanying this paper visit https://doi.org/10.1017/gmh.2022.26. click here to view supplementary material
