# Supplementary material for: Validating measures of stigma against those with mental illness among a community sample in Kilifi Kenya
Source: Glob Ment Health (Camb). 2022 Jun 3;9:241–8. doi: 10.1017/gmh.2022.26 (PMC9806973; doi:10.1017/gmh.2022.26)
Supplement: Supplementary file 1 [file S2054425122000267sup001.docx]

**Supplementary table 1.** Distribution of summated mean scores and standard deviations by sociodemographic variables, N=616

| Participant characteristic | Total RIBS score | Total MAKS score | CAMI factor 1 | CAMI factor 2 | CAMI factor 3 |
| --- | --- | --- | --- | --- | --- |
| Male, n=303 | 16.70 (4.27) | 44.35 (4.22) | 25.99 (6.81) | 31.36 (2.95) | 11.45 (2.50) |
| Female, n=313 | 16.06 (4.91) | 44.42 (4.06) | 28.06 (7.20) | 31.38 (3.17) | 11.58 (2.46) |
| P- value of between group differences using T-test | 0.29 | 0.99 | 0.00 | 0.15 | 0.20 |
| Level of education, n (%) | | | | |  |
| None, n=56 | 13.57 (5.35) | 42.63 (4.09) | 33.93 (6.78) | 30.93 (3.39) | 10.84 (2.88) |
| Primary, n=243 | 15.39 (5.07) | 42.76 (4.44) | 30.84 (6.57) | 31.03 (3.32) | 11.30 (2.50) |
| Secondary, n=221 | 15.84 (4.47) | 43.12 (4.29) | 26.69 (5.85) | 31.33 (2.93) | 11.23 (2.58) |
| Tertiary, n=96 | 16.90 (4.33) | 44.38 (4.36) | 24.35 (5.08) | 31.33 (2.91) | 11.40 (2.40) |
| P- value of between group differences | 0.00 | 0.02 | 0.00 | 0.65 | 0.60 |
| Experience with mental illness, n (%) either as a patient with psychiatric illness or epilepsy or as a caregiver of a patient with these disorders | | | | | |
| Yes, n=196 | 15.71 (5.03) | 43.78 (4.11) | 29.48 (6.60) | 31.54 (3.29) | 11.53 (2.51) |
| No, n=420 | 15.58 (4.75) | 42.83 (4.46) | 28.21 (6.83) | 31.01 (3.03) | 11.12 (2.56) |
| P- value of between group differences | 0.76 | 0.01 | 0.03 | 0.05 | 0.06 |

**Supplementary table 2.** Response frequencies for the RIBS scale, n=616

|  | Question | Yes n (%) | No n (%) | Don’t know n (%) | | |
| --- | --- | --- | --- | --- | --- | --- |
| 1 | Are you currently living with, or have you ever lived with, someone with a mental health problem? | 292 (47.4) | 315 (51.1) | 9 (1.5) | | |
| 2 | Are you currently working with, or have you ever worked with, someone with a mental health problem? | 109 (17.7) | 498 (80.8) | 9 (1.5) | | |
| 3 | Do you currently have, or have you ever had a neighbor with a mental health problem? | 383 (62.2) | 228 (37.0) | 5 (0.8) | | |
| 4 | Do you currently have, or have you ever had a close friend with a mental health problem? | 239 (38.8) | 370 (60.1) | 7 (1.1) | | |
|  |  | **Agree strongly** | **Agree slightly** | **Neither agree nor disagree/ Don’t Know** | **Disagree slightly** | **Disagree strongly** |
| 5 | In the future, I would be willing to live with someone with a mental health problem. | 336 (54.5) | 72 (11.7) | 90 (14.6) | 33 (5.4) | 85 (13.8) |
| 6 | In the future, I would be willing to work with someone with a mental health problem. | 278 (45.1) | 90 (14.6) | 89 (14.5) | 50 (8.1) | 109 (17.7) |
| 7 | In the future, I would be willing to live nearby to someone with a mental health problem. | 350 (56.8) | 78 (12.7) | 88 (14.3) | 26 (4.2) | 74 (12.0) |
| 8 | In the future, I would be willing to continue a relationship with a friend who developed a mental health problem. | 381 (61.9) | 89 (14.4) | 62 (10.1) | 24 (3.9) | 60 (9.7) |

**Supplementary table 3.** Response frequencies for the MAKS scale, n=616

|  | MAKS item | Responses, n (%) | | | | |
| --- | --- | --- | --- | --- | --- | --- |
|  |  | **Agree strongly** | **Agree slightly** | **Neither agree nor disagree/ Don’t know** | **Disagree slightly** | **Disagree strongly** |
| 1 | Most people with mental health problems want to have paid employment. | 207 (33.6) | 106 (17.2) | 75 (12.2) | 37 (6.0) | 191 (31.0) |
| 2 | If a friend had a mental health problem, I know what advice to give them to get professional help. | 444 (72.1) | 110 (17.8) | 42 (6.8) | 6 (1.0) | 14 (2.3) |
| 3 | Medication can be an effective treatment for people with mental health problems | 498 (80.8) | 78 (12.7) | 21 (3.4) | 10 (1.6) | 9 (1.5) |
| 4 | Psychotherapy (eg counselling or talking therapy) can be an effective treatment for people with mental health problems. | 377 (61.2) | 138 (22.4) | 40 (6.5) | 21 (3.4) | 40 (6.5) |
| 5 | People with severe mental health problems can fully recover. | 356 (57.8) | 133 (21.6) | 58 (9.4) | 34 (5.5) | 35 (5.7) |
| 6 | Most people with mental health problems go to a healthcare professional to get help. | 37 (6.0) | 38 (6.2) | 35 (5.7) | 104 (16.9) | 402 (65.2) |
|  | *For items 7-12, say whether you think each condition is a type of mental illness by ticking one box only.* | | | | | |
| 7 | Depression | 303 (49.2) | 84 (13.6) | 51 (8.3) | 36 (5.8) | 142 (23.1) |
| 8 | Stress | 103 (16.7) | 42 (6.8) | 45 (7.3) | 93 (15.1) | 333 (54.1) |
| 9 | Schizophrenia | 514 (83.4) | 48 (7.8) | 22 (3.6) | 4 (0.6) | 28 (4.6) |
| 10 | Bipolar disorder (manic depression) | 303 (49.2) | 85 (13.8) | 84 (13.6) | 52 (8.4) | 92 (14.9) |
| 11 | Drug addiction | 354 (57.5) | 62 (10.1) | 36 (5.8) | 46 (7.5) | 118 (19.1) |
| 12 | Grief | 152 (24.7) | 49 (7.9) | 53 (8.6) | 98 (15.9) | 264 (42.9) |

**Supplementary table 4.** Response frequencies for the CAMI scale, n=616

|  | CAMI item | Responses, n (%) | | | | |
| --- | --- | --- | --- | --- | --- | --- |
|  |  | **Strongly**  **agree** | **Agree** | **Neutral** | **Disagree** | **Strongly**  **disagree** |
|  | As soon as a person shows signs of mental disturbance, he should be hospitalized. | 266 (43.2) | 191 (31.0) | 28 (4.5) | 122 (19.8) | 9 (1.5) |
|  | More tax money should be spent on the care and treatment of the mentally ill. | 357 (58.0) | 209 (33.9) | 25 (4.1) | 23 (3.7) | 2 (0.3) |
|  | The mentally ill should be isolated from the rest of the community. | 22 (3.6) | 38 (6.2) | 14 (2.3) | 371 (60.2) | 171 (27.7) |
|  | The best therapy for many mental patients is to be part of a normal community. | 351 (57.0) | 203 (32.9) | 22 (3.6) | 34 (5.5) | 6 (1.0) |
|  | Mental illness is an illness like any other. | 176 (28.6) | 184 (29.9) | 23 (3.7) | 188 (30.5) | 45 (7.3) |
|  | The mentally ill are a burden on society. | 105 (17.1) | 127 (20.6) | 21 (3.4) | 267 (43.3) | 96 (15.6) |
|  | The mentally ill are far less of a danger than most people suppose. | 147 (23.9) | 220 (35.7) | 40 (6.5) | 172 (27.9) | 37 (6.0) |
|  | Locating mental health facilities in a residential area downgrades the neighbourhood. | 36 (5.8) | 58 (9.4) | 31 (5.0) | 364 (59.1) | 127 (20.6) |
|  | There is something about the mentally ill that makes it easy to tell them from normal people. | 272 (44.2) | 280 (45.4) | 25 (4.1) | 36 (5.8) | 3 (0.5) |
|  | The mentally ill have for too long been the subject of ridicule. | 261 (42.4) | 268 (43.5) | 24 (3.9) | 54 (8.8) | 9 (1.4) |
|  | A woman would be foolish to marry a man who has suffered from mental illness, even though he seems fully recovered. | 12 (2.0) | 51 (8.3) | 20 (3.2) | 360 (58.4) | 173 (28.1) |
|  | As far as possible mental health services should be provided through community- based facilities. | 359 (58.3) | 219 (35.5) | 3 (0.5) | 23 (3.7) | 12 (2.0) |
|  | Less emphasis should be placed on protecting the public from the mentally ill. | 260 (42.2) | 285 (46.3) | 20 (3.2) | 45 (7.3) | 6 (1.0) |
|  | Increased spending on mental health services is a waste of tax dollars. | 8 (1.3) | 24 (3.9) | 18 (2.9) | 405 (65.8) | 161 (26.1) |
|  | No one has the right to exclude the mentally ill from their neighbourhood. | 348 (56.5) | 234 (38.0) | 7 (1.1) | 21 (3.4) | 6 (1.0) |
|  | Having mental patients living within residential neighbourhoods might be good therapy, but the risks to residents are too great. | 166 (27.0) | 212 (34.4) | 44 (7.1) | 164 (26.6) | 30 (4.9) |
|  | Mental patients need the same kind of control and discipline as a young child. | 188 (30.5) | 237 (38.5) | 17 (2.8) | 148 (24.0) | 26 (4.2) |
|  | We need to adopt a far more tolerant attitude toward the mentally ill in our society. | 347 (56.3) | 255 (41.4) | 9 (1.5) | 5 (0.8) | - |
|  | I would not want to live next door to someone who has been mentally ill. | 8 (1.3) | 26 (4.2) | 22 (3.6) | 421 (68.3) | 139 (22.6) |
|  | Residents should accept the location of mental health facilities in their neighbourhood to serve the needs of the local community. | 313 (50.8) | 275 (44.6) | 9 (1.5) | 16 (2.6) | 3 (0.5) |
|  | The mentally ill should not be treated as outcasts of society. | 307 (49.8) | 282 (45.8) | 6 (1.0) | 17 (2.8) | 4 (0.6) |
|  | There are sufficient existing services for the mentally ill. | 139 (22.5) | 232 (37.7) | 53 (8.6) | 160 (26.0) | 32 (5.2) |
|  | Mental patients should be encouraged to assume the responsibilities of normal life. | 197 (32.0) | 257 (41.7) | 30 (4.9) | 115 (18.7) | 17 (2.7) |
|  | Local residents have good reason to resist the location of mental health services in their neighbourhood. | 57 (9.2) | 62 (10.1) | 26 (4.2) | 386 (62.7) | 85 (13.8) |
|  | The best way to handle the mentally ill is to keep them behind locked doors. | 14 (2.3) | 50 (8.1) | 15 (2.4) | 373 (60.6) | 164 (26.6) |
|  | Our mental hospitals seem more like prisons than like places where the mentally ill can be cared for. | 74 (12.0) | 118 (19.2) | 106 (17.2) | 268 (43.5) | 50 (8.1) |
|  | Anyone with a history of mental problems should be excluded from taking public office. | 14 (2.3) | 73 (11.8) | 25 (4.1) | 388 (63.0) | 116 (18.8) |
|  | Locating mental health services in residential neighbourhoods does not endanger local residents. | 218 (35.4) | 301 (48.9) | 21 (3.4) | 56 (9.1) | 20 (3.2) |
|  | Mental hospitals are an outdated means of treating the mentally ill. | 6 (1.0) | 32 (5.2) | 14 (2.3) | 413 (67.0) | 151 (24.5) |
|  | The mentally ill do not deserve our sympathy. | 10 (1.6) | 30 (4.9) | 5 (0.8) | 398 (64.6) | 173 (28.1) |
|  | The mentally ill should not be denied their individual rights. | 331 (53.7) | 263 (42.7) | 6 (1.0) | 13 (2.1) | 3 (0.5) |
|  | Mental health facilities should be kept out of residential neighbourhoods. | 43 (7.0) | 123 (20.0) | 21 (3.4) | 348 (56.5) | 81 (13.1) |
|  | One of the main causes of mental illness is a lack of self-discipline and will power. | 125 (20.3) | 165 (26.8) | 54 (8.8) | 222 (36.0) | 50 (8.1) |
|  | We have the responsibility to provide the best possible care for the mentally ill. | 381 (61.9) | 228 (37.0) | 4 (0.6) | 3 (0.5) |  |
|  | The mentally ill should not be given any responsibility. | 85 (13.8) | 172 (27.9) | 33 (5.4) | 269 (43.7) | 57 (9.2) |
|  | Residents have nothing to fear from people coming into their neighbourhood to obtain mental health services. | 257 (41.7) | 307 (49.8) | 21 (3.4) | 26 (4.2) | 5 (0.8) |
|  | Virtually anyone can become mentally ill. | 289 (46.9) | 261 (42.4) | 13 (2.1) | 46 (7.5) | 7 (1.1) |
|  | It is best to avoid anyone who has mental problems. | 16 (2.6) | 61 (9.9) | 13 (2.1) | 414 (67.2) | 112 (18.2) |
|  | Most women who were once patients in a mental hospital can be trusted as babysitters. | 135 (21.9) | 221 (35.9) | 62 (10.1) | 177 (28.7) | 21 (3.4) |
|  | It is frightening to think of people with mental problems living in residential neighbourhoods. | 80 (13.0) | 160 (26.0) | 47 (7.6) | 285 (46.3) | 44 (7.1) |


**Supplementary table 5.** Exploratory factor analysis results for the original CAMI scale

| Question | Factor | | | | | | Uniqueness |
| --- | --- | --- | --- | --- | --- | --- | --- |
|  | **1** | **2** | **3** | **4** | **5** | **6** |  |
| Mental hospitals are an outdated means of treating the mentally ill | 0.64 | 0.09 | 0.18 | 0.03 | 0.14 | 0.00 | 0.73 |
| The best way to handle the mentally ill is to keep them behind locked doors | 0.60 | -0.05 | -0.06 | -0.06 | 0.10 | 0.02 | 0.74 |
| I would not want to live next door to someone who has been mentally ill | 0.60 | -0.08 | 0.10 | 0.02 | 0.00 | -0.04 | 0.63 |
| The mentally ill do not deserve our sympathy | 0.59 | -0.09 | 0.07 | 0.11 | -0.14 | 0.00 | 0.68 |
| It is best to avoid anyone who has mental problems | 0.53 | -0.02 | -0.14 | -0.06 | 0.02 | -0.02 | 0.70 |
| Increased spending on mental health services is a waste of tax dollars | 0.53 | -0.14 | 0.10 | 0.14 | -0.05 | -0.00 | 0.65 |
| The mentally ill should be isolated from the rest of the community | 0.46 | -0.05 | 0.01 | -0.03 | 0.20 | 0.04 | 0.70 |
| A woman would be foolish to marry a man who has suffered from mental illness, even though he seems fully recovered | 0.45 | -0.11 | -0.02 | 0.09 | 0.06 | 0.17 | 0.60 |
| Locating mental health facilities in a residential area downgrades the neighbourhood | 0.42 | -0.06 | 0.02 | 0.12 | 0.06 | 0.16 | 0.77 |
| One of the main causes of mental illness is a lack of self-discipline and will power | 0.41 | 0.13 | 0.05 | -0.00 | 0.09 | -0.27 | 0.75 |
| Anyone with a history of mental problems should be excluded from taking public office | 0.40 | -0.11 | -0.10 | -0.12 | 0.24 | 0.07 | 0.63 |
| Mental health facilities should be kept out of residential neighbourhoods | 0.39 | 0.11 | -0.29 | -0.15 | 0.08 | -0.10 | 0.78 |
| The mentally ill should not be given any responsibility | 0.34 | 0.03 | -0.18 | 0.07 | 0.08 | -0.15 | 0.67 |
| It is frightening to think of people with mental problems living in residential neighbourhoods | 0.30 | 0.15 | -0.11 | 0.03 | 0.07 | -0.02 | 0.54 |
| We need to adopt a far more tolerant attitude toward the mentally ill in our society | -0.10 | 0.66 | 0.06 | -0.02 | 0.05 | 0.10 | 0.66 |
| The mentally ill should not be denied their individual rights | -0.05 | 0.66 | -0.10 | 0.04 | -0.18 | -0.03 | 0.69 |
| The mentally ill should not be treated as outcasts of society | -0.00 | 0.63 | 0.04 | -0.01 | -0.00 | 0.03 | 0.79 |
| We have the responsibility to provide the best possible care for the mentally ill | -0.17 | 0.61 | 0.03 | 0.01 | 0.01 | 0.04 | 0.56 |
| Residents should accept the location of mental health facilities in their neighbourhood to serve the needs of the local community | -0.07 | 0.55 | 0.05 | 0.05 | -0.00 | 0.02 | 0.56 |
| No one has the right to exclude the mentally ill from their neighbourhood | -0.11 | 0.42 | 0.05 | 0.07 | 0.14 | -0.03 | 0.62 |
| Residents have nothing to fear from people coming into their neighbourhood to obtain mental health services | -0.01 | 0.38 | 0.10 | 0.00 | 0.02 | -0.20 | 0.53 |
| Locating mental health services in residential neighbourhoods does not endanger local residents | 0.06 | 0.27 | 0.14 | -0.04 | -0.19 | -0.24 | 0.68 |
| As far as possible mental health services should be provided through community- based facilities | -0.02 | 0.23 | 0.04 | 0.21 | 0.18 | -0.07 | 0.71 |
| Having mental patients living within residential neighbourhoods might be good therapy, but the risks to residents are too great | 0.20 | 0.23 | -0.20 | 0.21 | 0.08 | -0.20 | 0.73 |
| The mentally ill are far less of a danger than most people suppose | 0.08 | 0.07 | 0.53 | -0.09 | 0.04 | -0.01 | 0.54 |
| Mental illness is an illness like any other | 0.09 | 0.03 | 0.52 | -0.08 | 0.02 | 0.02 | 0.78 |
| The best therapy for many mental patients is to be part of a normal community | 0.01 | 0.11 | 0.41 | 0.27 | -0.01 | -0.08 | 0.64 |
| Mental patients should be encouraged to assume the responsibilities of normal life | 0.14 | 0.20 | 0.30 | 0.24 | 0.05 | 0.24 | 0.74 |
| Virtually anyone can become mentally ill | -0.01 | 0.23 | 0.29 | -0.19 | 0.17 | -0.02 | 0.52 |
| More tax money should be spent on the care and treatment of the mentally ill | -0.08 | -0.01 | 0.28 | 0.23 | 0.25 | -0.14 | 0.54 |
| There are sufficient existing services for the mentally ill | 0.16 | 0.05 | -0.05 | 0.48 | -0.08 | 0.13 | 0.59 |
| Less emphasis should be placed on protecting the public from the mentally ill | 0.07 | 0.34 | -0.06 | 0.41 | 0.09 | 0.02 | 0.68 |
| As soon as a person shows signs of mental disturbance, he should be hospitalized | 0.06 | -0.11 | -0.01 | 0.31 | 0.29 | 0.04 | 0.73 |
| Our mental hospitals seem more like prisons than like places where the mentally ill can be cared for | 0.29 | 0.08 | -0.03 | -0.30 | 0.01 | 0.05 | 0.61 |
| The mentally ill have for too long been the subject of ridicule | -0.02 | 0.16 | 0.17 | -0.14 | 0.39 | -0.00 | 0.67 |
| The mentally ill are a burden on society | 0.34 | -0.00 | -0.06 | 0.06 | 0.37 | 0.01 | 0.67 |
| There is something about the mentally ill that makes it easy to tell them from normal people | -0.07 | 0.28 | 0.05 | 0.19 | 0.29 | -0.06 | 0.72 |
| Mental patients need the same kind of control and discipline as a young child | 0.11 | 0.14 | 0.03 | 0.09 | 0.03 | 0.38 | 0.56 |
| Local residents have good reason to resist the location of mental health services in their neighbourhood | 0.28 | 0.14 | -0.05 | 0.09 | -0.06 | 0.33 | 0.83 |
| Most women who were once patients in a mental hospital can be trusted as babysitters | 0.01 | 0.14 | 0.15 | 0.12 | 0.03 | -0.01 | 0.75 |

The 23 items marked in red were selected for inclusion in the CFA using following criteria (Norris and Lecavalier, 2010, 2008): i) each factor contained only items that explained ≥ 10% of the factor’s variance, (ii) factors had at least 3 items loading with a factor loading ≥ 0.32 (iii) only items that did not cross load on multiple factors with similar magnitudes were extracted and (iv) factors were interpretable in a contextually sensible way.


**Supplementary file 1**. Kiswahili version of the RIBS scale

**VIPIMO VYA TABIA VILIVYORIPOTIWA NA KUKUSUDIWA RIBS**

**Maagizo**: Maswali yafuatayo yanaulizia kuhusu uzoefu wako na maoni yako kuhusiana na watu walio na matatizo ya afya ya kiakili [kwa mfano, watu walioonekana na wahudumu wa afya]. Kwa kila swali 1-4, tafadhali jibu ukitumia majibu yafuatayo: **[MSOMEE MSHIRIKI MAJIBU]**

|  | | **Ndiyo** | **La** | **Sijui** |
| --- | --- | --- | --- | --- |
| 1 | Je, kwa hivi sasa unaishi, au ushawahi ishi na mtu mwenye matatizo ya afya ya kiakili? [matatizo ya kiakili] |  |  |  |
| 2 | Je, kwa hivi sasa unafanya kazi au ushawahi kufanya kazi na mtu mwenye matatizo ya afya ya kiakili? [matatizo ya kiakili] |  |  |  |
| 3 | Je, kwa hivi sasa uko na jirani au ushawahi kuwa na jirani mwenye matatizo ya afya ya kiakili? [matatizo kiakili] |  |  |  |
| 4 | Je, kwa hivi sasa uko na rafiki au ushawahi kuwa na rafiki aliye na matatizo ya afya ya kiakili? [matatizo ya kiakili] |  |  |  |

**Maagizo**: Kwa kila kauli 5-8, jibu kwa kuchangua mojawapo ya majibu yafuatayo: **[MSOMEE MSHIRIKI MAJIBU]**

|  | |  |  |  |  |  |  |
| --- | --- | --- | --- | --- | --- | --- | --- |
|  |  | **Nakubaliana kabisa** | **Nakubaliana kidogo** | **Sikubali wala sikatai** | **NaKataa kidogo** | **Nakataa kabisa** | **Sijui** |
| 5 | Katika siku za usoni, nitakuwa tayari kuishi na mtu mwenye matatizo ya afya ya kiakili [matatizo ya kiakili] |  |  |  |  |  |  |
| 6 | Katika siku za usoni, nitakuwa tayari kufanya kazi na mtu mwenye matatizo ya afya ya kiakili [matatizo ya kiakili] |  |  |  |  |  |  |
| 7 | Katika siku za usoni, nitakuwa tayari kuishi karibu na mtu mwenye matatizo ya afya ya kiakili [matatizo ya kiakili] |  |  |  |  |  |  |
| 8 | Katika siku za usoni, nitakuwa tayari kuendeleza uhusiano na rafiki aliyepatwa na matatizo ya afya ya kiakili [matatizo ya kiakili] |  |  |  |  |  |  |

**Ahsante sana kwa msaada wako.**

Vipimo vya Tabia vilivyoripotiwa na kukusudiwa **RIBS** 10 © **2009** Kitengo cha Utafiti cha iadi ya watu na huduma za afya, Taasisi ya Matatizo ya akili, Chuo Kikuu cha King’s, London. Mawasiiano: Profesa Graham Thornicroft. Barua pepe: [graham.thornicroft@kcl.ac.uk](mailto:graham.thornicroft@kcl.ac.uk)

**Muda wa mahojiano:** Dakika………………… Sekunde…………………………...

**Supplementary file 2.** Kiswahili version of the MAKS scale

**RATIBA YA UFAHAMU WA AFYA YA AKILI** **MAKS**

**Maagizo:** Kwa kila kauli 1-6, jibu kwa kuchagua mojawapo ya majibu haya **[MSOMEE MSHIRIKI MAJIBU].** Matatizo ya afya ya akili hapa yanamaanisha, matatizo au (kwa mfano), hali ambazo zinaweza zikamfanya mtu aonekane na muhudumu wa afya.

|  |  | **Nakubaliana kabisa** | **Nakubaliana kidogo** | **Sikubali wala sikatai** | **NaKataa kidogo** | **Nakataa kabisa** | **Sijui** |
| --- | --- | --- | --- | --- | --- | --- | --- |
| 1 | Watu wengi walio na matatizo ya afya ya kiakili /matatizo ya kiakili wanataka kuajiriwa. |  |  |  |  |  |  |
| 2 | Ikiwa rafiki yuko na matatizo ya afya ya kiakili/matatizo ya kiakili, najua ushauri wa kumpa ili apate usaidizi wa kitaalamu. |  |  |  |  |  |  |
| 3 | Kutumia dawa inaweza kuwa njia mwafaka ya matibabu kwa watu walio na matatizo ya afya ya kiakili/matatizo ya kiakili. |  |  |  |  |  |  |
| 4 | Tiba ya kisaikolojia [kwa mfano, ushauri ama tiba ya mazungumzo] inaweza kuwa tiba mwafaka kwa watu walio na matatizo ya afya ya kiakili/matatizo ya kiakili. |  |  |  |  |  |  |
| 5 | Watu wenye matatizo makali ya afya ya kiakili /ya kiakili wanaweza kupona kabisa. |  |  |  |  |  |  |
| 6 | Watu wengi walio na matatizo ya afya ya kiakili /matatizo ya kiakili huenda kwa wataalamu wa afya ili kupata usaidizi. |  |  |  |  |  |  |
| **Maagizo**: Kwa maswali 7-12, sema kama kila hali ni aina ya magonjwa ya akili kwa kuchagua mojawapo ya majibu haya **[MSOMEE MSHIRIKI MAJIBU]** | | | | | | | |
| 7 | Huzuni (Sonona)  ***(Maelezo: Hii ni huzuni kupita kiasi,inayoendelea kwa zaidi ya wiki mbili mfululizo na inayomfanya mtu asiweze kuendelea na shughuli zake za kila siku)*** |  |  |  |  |  |  |
| 8 | Msongo wa mawazo |  |  |  |  |  |  |
| 9 | Skizofrenia/Wazimu |  |  |  |  |  |  |
|  |  | **Nakubaliana kabisa** | **Nakubaliana kidogo** | **Sikubali wala sikatai** | **NaKataa kidogo** | **Nakataa kabisa** | **Sijui** |
| 10 | Maradhi ya hisia mseto[unyogovu wa manic]  ***(Maelezo: hii ni hali ambayo humfanya mtu kuwa na furaha nyingi kupita kiasi bila ya sababu maalum inayofuatwa na huzuni nyingi kupita kiasi bila ya sababu maalum. Hali hizi mbili hufuatana na wakati mwingine hubadilisha utendaji kazi wa mwadhiriwa)*** |  |  |  |  |  |  |
| 11 | Uraibu wa dawa/ madawa ya kulevya |  |  |  |  |  |  |
| 12 | Majonzi |  |  |  |  |  |  |

**Ahsante sana kwa msaada wako.**

Ratiba ya ufahamu wa afya ya akili MAKS 10 © 2009 kitengo cha utafiti cha huduma za afya na idadi ya watu, Taasisi ya magonjwa ya akili , Chuo Kikuu cha King’s Collection. Mawasiliano: Profesa Graham Thornicroft. Barua pepe: [graham.thornicroft@kcl.ac.uk](mailto:graham.thornicroft@kcl.ac.uk)

Muda wa mahojiano: Dakika………………… Sekunde…………………………...

**Supplementary file 3.** Kiswahili version of the CAMI scale

**MITAZAMO YA JAMII KWA WAGONJWA WA KIAKILI CAMI**

**Maagizo:** Kauli zifuatazo zinaelezea maoni mbalimbali kuhusu ugonjwa wa akili na wanaougua ugonjwa wa kiakili. Wanaougua ugonjwa wa kiakili ni watu wanaohitaji matibabu kwa ajili ya matatizo ya kiakili lakini wanaweza kujitegemea wenyewe kuishi nje ya hospitali. Tafadhali chagua jibu ambalo linaelezea kwa usahihi mtazamo wako kwa kila kauli. Ni jibu lako la kwanza ndilo lenye umuhimu zaidi. Usiwe na shaka ikiwa kauli nyingine zinakaa kufanana na kauli ulizojibu mwanzoni. Tafadhali hakikisha unajibu kauli zote

|  |  | **SA=**  **Nakubaliana kabisa** | **A=**  **Nakubuliana** | **N=**  **Sina la Kusema/Sijui** | **D=**  **Napinga** | **SD=**  **Napinga kabisa** |
| --- | --- | --- | --- | --- | --- | --- |
| a | Punde tu mtu anapoonyesha dalili za matatizo ya kiakili anafaa kulazwa hospitalini. |  |  |  |  |  |
| b | Pesa zaidi za ushuru sharti zitumike kwa huduma na matibabu ya wagonjwa wa kiakili. |  |  |  |  |  |
| c | Wagonjwa wa kiakili sharti watengwe na jamii. |  |  |  |  |  |
| d | Tiba bora kwa wagonjwa wengi wa kiakili ni kuwa pamoja na jamii ya kawaida. |  |  |  |  |  |
| e | Ugonjwa wa kiakili ni sawa tu na magonjwa mengine. |  |  |  |  |  |
| f | Wagonjwa wa kiakili ni mzigo kwa jamii. |  |  |  |  |  |
| g | Wagonjwa wa kiakili si hatari sana kama vile watu wengi hudhania. |  |  |  |  |  |
| h | Kuweka vituo vya afya ya kiakili katika eneo la makaazi inashusha hadhi ya kijiji. |  |  |  |  |  |
| i | Kuna dalili flani kuhusu wagonjwa wa kiakili ambazo zinarahisisha kuwatofautisha na watu wa kawaida. |  |  |  |  |  |
| j | Wagonjwa wa kiakili kwa mda mrefu wamekuwa wakidhihakiwa / wakidharauliwa. |  |  |  |  |  |
| k | Mwanamke atakuwa mpumbavu kuolewa na mwanamume ambaye aliugua ugonjwa wa kiakili ingawa amepona kabisa. |  |  |  |  |  |
| l | Kwa vyovyote vile huduma za matatizo ya kiakili sharti zipeanwe katika vituo vya afya vya nyanjani. |  |  |  |  |  |
| m | Mkazo kidogo unafaa uwekwe kwa kulinda umma kutokana na wagonjwa wa kiakili. |  |  |  |  |  |
| n | Ongezeko la matumizi ya fedha kwa huduma za afya ya kiakili ni kupoteza pesa za ushuru. |  |  |  |  |  |
| o | Hakuna aliye na haki ya kumtenga mgonjwa wa kiakili kutoka kijijini au kwa jamii. |  |  |  |  |  |
| p | Kuishi na wagonjwa ya kiakili katika jamii inaweza kuwa tiba nzuri kwao, ila madhara kwa jamii yanaweza kuwa makuu zaidi. |  |  |  |  |  |
| q | Wagonjwa wa kiakili wanahitaji kuthibitiwa na kupewa nidhamu kama vile mtoto mdogo. |  |  |  |  |  |
| r | Tunahitaji tujifunze kuwa na dhana ya uvumilivu zaidi kwa wanaouguwa matatizo ya kiakili katika jamii. |  |  |  |  |  |
| s | Singependa kuwa jirani wa karibu na mtu ambaye amekuwa akiugua ugonjwa ya kiakili. |  |  |  |  |  |
| t | Wakaazi sharti wakubali kuwekewa vituo vya afya ya kiakili kwenye vijiji vyao ili kuhudumia mahitaji yao. |  |  |  |  |  |
| u | Wagonjwa wa kiakili wasichukuliwe kama waliotengwa katika jamii. |  |  |  |  |  |
| v | Kuna huduma za kutosha zilizopo kwa wagonjwa wa kiakili. |  |  |  |  |  |
| w | Wagonjwa wa kiakili sharti wahimizwe kuchukua majukumu ya kawaida ya maisha. |  |  |  |  |  |
| x | Wakaazi wana sababu mwafaka ya kupinga kuwepo kwa kituo cha huduma za afya ya kiakili katika kijiji chao. |  |  |  |  |  |
| y | Njia nzuri ya kuwashughulikia wagonjwa wa kiakili ni kuwafungia. |  |  |  |  |  |
| z | Hospitali zetu za kiakili zinafanana zaidi na magereza kuliko kama mahali ambapo wagonjwa wa kiakili wanaweza kuhudumiwa. |  |  |  |  |  |
| aa | Mtu yeyote aliye na historia ya matatizo ya kiakili sharti atengwe kutokana na kuajiriwa kwa ofisi za umma. |  |  |  |  |  |
| bb | Kuweka vituo vya huduma za afya ya kiakili katika vijiji hakuwahatarishi wakaazi. |  |  |  |  |  |
| cc | Hospitali za wagonjwa wa kiakili ni njia zilizopitwa na wakati kwa kuwatibu wagonjwa wa kiakili. |  |  |  |  |  |
| dd | Wagonjwa wa kiakili hawastahili huruma zetu. |  |  |  |  |  |
| ee | Wagonjwa wa kiakili wasinyimwe haki zao. |  |  |  |  |  |
| ff | Vituo vya huduma za afya ya kiakili vinafaa viwekwe mbali na makaazi ya watu. |  |  |  |  |  |
| gg | Mojawapo ya sababu kuu za magonjwa ya kiakili ni ukosefu wa nidhamu binafsi na uwezo wa kujizuia kufanya kitu. |  |  |  |  |  |
| hh | Tuko na jukumu la kuwapatia wagonjwa wa kiakili huduma bora zaidi iwezekanavyo. |  |  |  |  |  |
| ii | Wagonjwa wa kiakili wasipewe jukumu lolote. |  |  |  |  |  |
| jj | Wakaazi hawana chochote cha kuogopa kwa watu wanaokuja katika kijiji chao ili kupata huduma za afya ya kiakili. |  |  |  |  |  |
| kk | Mtu yeyote anaweza kuugua ugonjwa wa kiakili. |  |  |  |  |  |
| ll | Ni bora kumuepuka mtu yeyote aliye na matatizo ya kiakili. |  |  |  |  |  |
| mm | Wanawake wengi ambao awali wamewahi kuuguzwa katika hospital za kiakili wanaweza kuaminika kuwa watunza watoto. |  |  |  |  |  |
| nn | Inaogopesha ukifikiria kuwa watu wenye matatizo ya kiakili wanaishi vijijini. |  |  |  |  |  |

Muda wa mahojiano: Dakika………………… Sekunde…………………………...
